# The integration effectiveness of inclusive sports policies: institutional support and community network mediation for sports participation of adults with disabilities and older adults in Chengdu under social capital theory

**DOI:** 10.3389/fpubh.2026.1881223

**Published:** 2026-09-10

**Authors:** Yufei Liu, Yiwen Wang

**Affiliations:** School of Physical Education, Suqian University, Suqian, Jiangsu, China

**Keywords:** community network, inclusive sports policy, marginalized groups, participatory governance, policy tools, social capital

## Abstract

**Introduction:**

Adults with Disabilities and Older Adults face unequal access to sports facilities, services, and policy information. In Chengdu, these barriers are linked to uneven resource allocation, limited accessibility, and differences in community organizational capacity and institutional trust. Drawing on social capital theory, this study examines how supply-side and demand-side inclusive sports policies are associated with sports participation through structural capital, relational capital, and community-network functions.

**Methods:**

A cross-sectional survey was conducted among 752 Adults with Disabilities and Older Adults in Chengdu. Institutional support was measured through supply-side and demand-side policy indicators derived from municipal policy documents and survey responses. Structural capital reflected community sports-organization density and activity frequency, while relational capital represented institutional trust. Community-network functions included resource matching and information transmission. Sports participation was assessed using a composite index of frequency, duration, and activity diversity. Structural equation modeling, hierarchical regression, and 5,000-sample bootstrap analyses were used to test mediation, moderation, and subgroup differences.

**Results:**

The model showed acceptable fit (χ^2^/df = 2.87, CFI = 0.937, TLI = 0.921, RMSEA = 0.048, SRMR = 0.041). Supply-side policy tools were positively associated with structural capital (*β* = 0.41, *p* < 0.001), which was associated with resource matching (*β* = 0.52). The indirect pathway from supply-side policy through structural capital and resource matching to sports participation was significant (effect = 0.066, 95% CI [0.042, 0.091]). Demand-side policy was positively associated with relational capital, and relational capital strengthened its association with participation (interaction *β* = 0.17, *p* < 0.01). The indirect pathway through relational capital and information transmission was significant (effect = 0.065, 95% CI [0.039, 0.090]). Older Adults appeared more sensitive to institutional trust, whereas Adults with Disabilities showed stronger dependence on resource matching.

**Discussion:**

Inclusive sports policies are associated with participation through different mechanisms. Supply-side policies rely more on community organizations and resource matching, while demand-side on trust and information. Policy design should move beyond uniform resource provision and address group-specific accessibility and communication needs. Because the study used cross-sectional data from Chengdu, the findings should not be interpreted as causal and require longitudinal and comparative verification.

## Introduction

Globally, marginalized populations continue to face structural barriers to sports participation, making inclusive access to physical activity an important issue for social equity and sustainable development (1). Economically disadvantaged groups, rural residents, Older Adults, and Adults with Disabilities often experience unequal access to facilities, services, information, and policy support. These inequalities may be intensified by urbanization and spatially uneven resource allocation. As sports services form part of basic public services, disparities in sports accessibility have become an important indicator of social inclusion (2, 3). Although many developed countries established relatively broad public sports systems earlier, participation gaps remain among low-income communities and minority populations. Developing countries additionally face the coexistence of rural resource shortages and inadequate services in urban fringe communities (4).

Existing policy practice generally relies on supply-side, demand-side, and environmental tools. Supply-side policies increase facilities, equipment, instructors, and service resources, but additional resources do not necessarily produce stable participation when community organizations lack the capacity to operate programs continuously (5–7). Demand-side policies, including subsidies, sports vouchers, and targeted assistance, may improve the precision of support, but their effectiveness depends on whether target groups can obtain and understand policy information and trust application and allocation procedures (8). Environmental tools can create a supportive atmosphere through regulations and public communication, but their effects are usually indirect. The central policy problem is therefore not simply the shortage of one type of instrument, but the lack of effective matching between policy tools and the organizational and relational conditions of communities (9).

**Figure 1:**
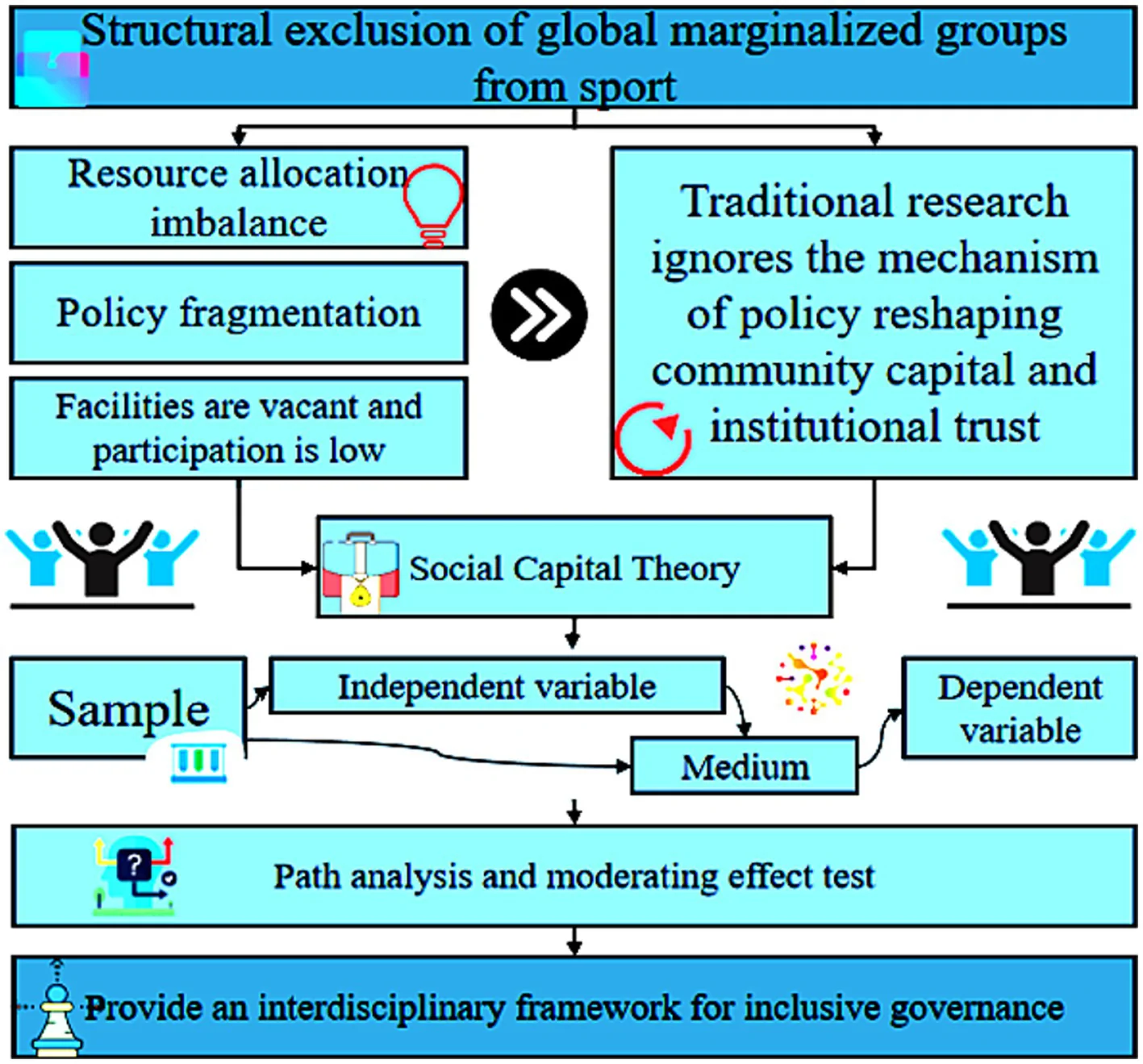
Article structure diagram.

Sports marginalization is multidimensional rather than purely economic (10, 11). Income constraints, spatial isolation, physical limitations, life-course needs, information capacity, and social relationships may overlap and shape whether formally available services can actually be used (12, 13). However, the empirical scope of this study is deliberately limited. It includes only Adults with Disabilities certified by the Chengdu Disabled Persons’ Federation and Older Adults who are permanent Chengdu residents aged 60 years or older. The findings should not be generalized to economically disadvantaged people without disabilities, geographically isolated populations outside Chengdu, migrant children, or other groups not represented in the sample (14) (Figure 1).

Previous studies show that the relationship between facility provision and inclusive sports participation is not linear. Research on disability sport indicates that inclusion depends not only on the existence of venues, but also on accessible environments, professional support, organizational acceptance, appropriate entry arrangements, and the removal of discriminatory barriers (15, 16). These findings suggest that the key question for supply-side policy is whether newly provided resources can be activated and converted into sustained participation opportunities. Research on demand-side tools likewise shows that subsidies and targeted support may fail when information channels are weak, administrative procedures are difficult to understand, or residents have limited trust in policy implementation (17, 18).

Community sport research further suggests that local organizations and support networks can reduce participation barriers and strengthen social inclusion (19). Social capital is therefore relevant, but it should not be treated as a single undifferentiated resource. Structural capital reflects the density and sustained activity capacity of community sports organizations. Such organizations may integrate facilities, personnel, and services, reduce participation costs, and organize regular activities (20). However, organizational density alone does not guarantee participation when organizations lack professional management or stable funding (21). Relational capital reflects residents’ trust in policy implementation, fair resource allocation, and grassroots governance. Higher trust may improve cooperation with targeted programs, whereas low trust may discourage eligible groups from applying for or using support (22).

Community networks provide the functional channels through which social capital is converted into practical outcomes. Resource matching concerns the connection between residents’ needs and accessible facilities or services, while information transmission concerns the reach, clarity, and credibility of policy information. Limited channels may produce information delay or distortion, whereas fragmented or highly digitalized channels may exclude people with weak digital literacy. Older Adults may depend more strongly on familiar interpersonal networks, while Adults with Disabilities may be more constrained by physical accessibility and specialized facility requirements (23, 24). International evidence also remains segmented: disability research mainly emphasizes accessibility and discrimination, while research on Older Adults gives greater attention to community participation, social connections, social support, physical activity, and health outcomes (25, 26). It is therefore uncertain whether the two groups share the same policy transmission mechanisms.

The remaining research gap concerns the differentiated functions of policy tools, social capital, and community networks. Existing studies rarely test whether supply-side and demand-side policies operate through different forms of social capital and different community network functions within one analytical framework. This study addresses that gap by distinguishing supply-side from demand-side policies, structural from relational capital, and resource matching from information transmission. It makes three contributions. First, it links structural capital primarily to the transformation of supply-side resources and relational capital to the acceptance of demand-side support. Second, it separates community network into resource matching and information transmission rather than treating it as a single mediator. Third, it tests moderated and chain-mediation associations to identify which form of social capital matters for which policy pathway. Because the study uses cross-sectional data, these relationships are interpreted as theoretically consistent statistical associations rather than confirmed causal sequences.

Based on the above framework, institutional support, social capital, and community network are treated as distinct but connected analytical levels. Supply-side policies improve the availability of facilities, equipment, instructors, and services, but their conversion into participation opportunities depends on the coordinating capacity of community organizations. Demand-side policies improve access to subsidies and targeted services, but their acceptance depends on credible information and institutional trust (27, 28). Structural capital is therefore expected to support resource matching, whereas relational capital is expected to strengthen information transmission. The following hypotheses test these differentiated associations without assuming a confirmed temporal or causal order (29):

*H1*: Supply-side policy tools are positively associated with sports participation among Adults with Disabilities and Older Adults in Chengdu through enhanced structural capital (community sports organization density).

*H2*: Relational capital (institutional trust) positively moderates the association between demand-side policy tools and sports participation.

*H3*: Community network functions, including resource matching and information transmission, form chain-mediating pathways between social capital and sports participation.

## Definition of terms

Inclusive sports policies refer to government-led arrangements intended to reduce institutional, environmental, informational, and social barriers to sports participation. In this study, the term is applied specifically to policies affecting Adults with Disabilities and Older Adults and is informed by research that treats inclusion as active participation supported by appropriate environments, organizations, and opportunities (30, 31).

Institutional support refers to public policy instruments and resources initiated or coordinated by government authorities. Supply-side support includes facilities, equipment, venue renovation, instructors, and service capacity, whereas demand-side support includes subsidies, sports vouchers, targeted assistance, and service purchasing. This distinction follows established policy-instrument classifications while recognizing that effective governance normally requires a coordinated instrument mix (32, 33).

Social capital refers to organizational connections and trust-based relationships that support collective action. Structural capital denotes the density and sustained activity capacity of community sports organizations. Relational capital denotes residents’ trust in policy implementation, resource allocation, and grassroots governance (34).

Community network refers to the functional connections through which community actors coordinate services and transmit policy information. It is operationalized as resource matching, which concerns the accessibility and suitability of facilities and services, and information transmission, which concerns the availability, clarity, and credibility of policy information (35).

Sports participation refers to respondents’ actual engagement in sport and physical activity. The study uses a standardized composite index based on participation frequency, duration per session, and diversity of sport types, consistent with widely used physical-activity dimensions and measurement practice (36, 37).

The term Adults with Disabilities refers to adults whose disability status was certified by the Chengdu Disabled Persons’ Federation. The term Older Adults refers to permanent Chengdu residents aged 60 years or older. References to marginalized groups in the theoretical background are broader, but all empirical findings and interpretations in this study are limited to these two sampled populations.

## Research design

### Variable selection

The independent variable is institutional support, measured through supply-side and demand-side policy tools. Supply-side intensity was derived from 12 Chengdu municipal policy documents (2020–2024) using TF-IDF scores for five keywords: facility construction, equipment provision, talent cultivation, venue renovation, and sports instructors. Demand-side tools were measured via a two-step approach: first, TF-IDF of five keywords (consumption subsidies, targeted assistance, service purchases, sports vouchers, means-tested support) from the same corpus; second, individual-level survey items (five-point Likert) capturing awareness of vouchers, participation in targeted subsidies, and perceived policy accuracy. A composite demand index was built as 0.4 × Z (text) + 0.6 × Z (survey), weighting determined by an expert panel. Sensitivity analyses with 0.5/0.5 and 0.3/0.7 weights gave consistent results (38). The dependent variable is sports participation of marginalized groups (economically, geographically, or age-disadvantaged). Three dimensions were standardized and summed: frequency (days/week from CHNS), intensity (IPAQ duration/session), and diversity (number of sport types) (39). The mediator is community network. Resource matching was measured as walking time (minutes) from home to the nearest public sports venue via map API. Information transmission used two items: channels of policy information and clarity on applying for subsidies, based on community service guidelines (40). The moderator is social capital, split into structural and relational capital. Structural capital used census data (community sports organizations per 10,000 people) plus monthly community activity frequency. Relational capital employed the CFPS government trust scale (five items, e.g., satisfaction with local sports policy implementation; total score 5–25) (41). The control variables include age, gender, education level, and monthly per capita household income.

**Figure 2:**
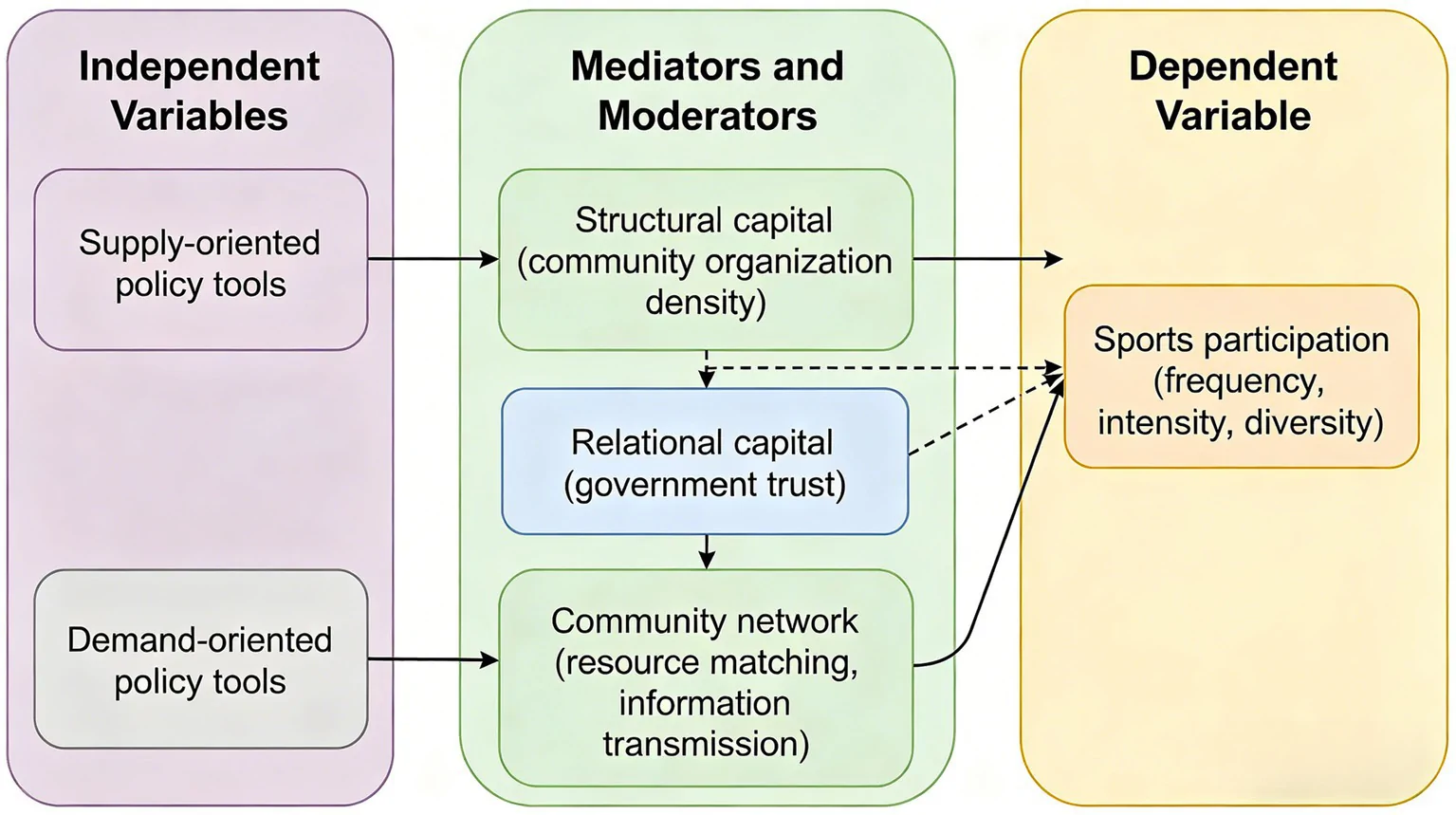
Variable design.

In summary, the variable selection for this study is illustrated in Figure 2.

### Research design

**Figure 3:**
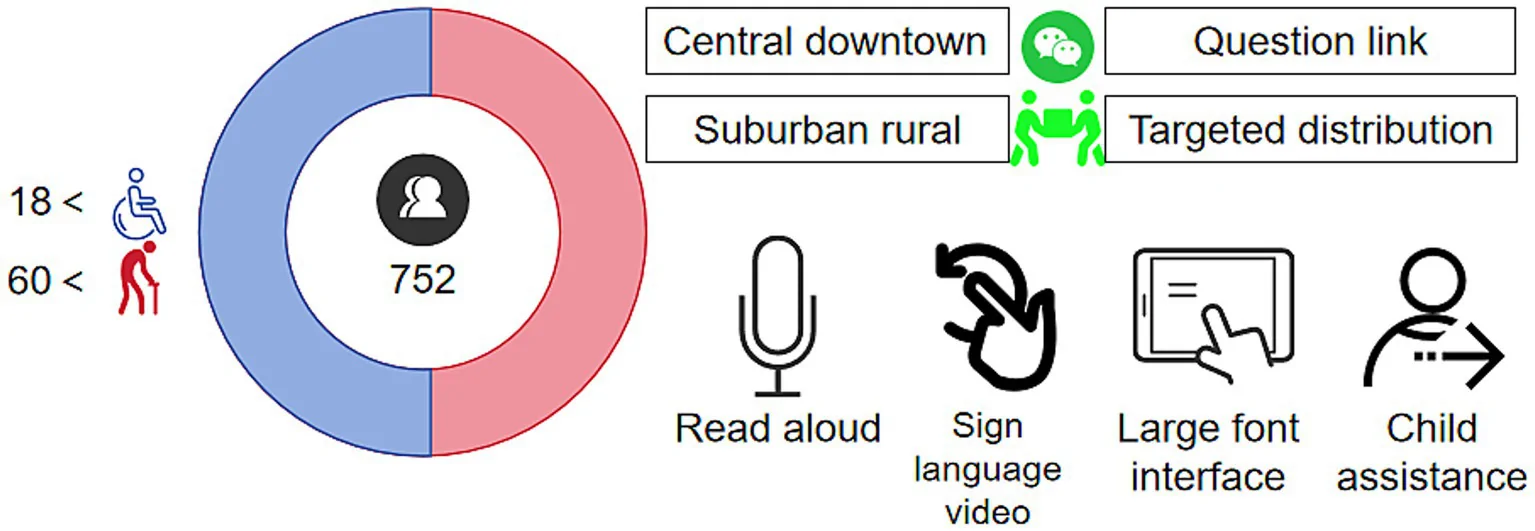
Research design.

This study adopts a cross-sectional survey design. *A priori* power analysis using G*Power 3.1 with a medium association size of f^2^ = 0.15, *α* = 0.05, power = 0.80, and up to 15 predictors indicated a minimum required sample of 139 cases. To account for possible design effects, incomplete questionnaires, and sample attrition, 800 questionnaires were distributed. After invalid and incomplete responses were excluded, 752 valid questionnaires were retained, exceeding the minimum sample size requirement (see Figure 3).

Seven research tools were used in this study, and each was assessed separately for reliability and validity. The supply side policy tool is measured by TF-IDF text analysis of 12 Chengdu Municipal policy documents from 2020 to 2024. This time window is used to characterize the formal policy environment of this research sample, and is not regarded as a complete record of long-term policy evolution or continuous implementation effect. The coding focused on five keywords: facility construction, equipment provision, talent cultivation, venue renovation, and sports instructors. Inter-coder reliability was acceptable, with Kappa = 0.88, and content validity was confirmed with CVI = 0.91.

Demand-side policy tools were measured using a composite index combining policy-text analysis and individual survey responses. The policy-text component used TF-IDF scores for five keywords: consumption subsidies, targeted assistance, service purchases, sports vouchers, and means-tested support. The survey component included three five-point Likert items measuring awareness of sports vouchers, participation in targeted subsidies, and perceived policy accuracy. The composite demand-side index was calculated as 0.4 × standardized text score + 0.6 × standardized survey score. Reliability and validity were acceptable, with Cronbach’s *α* = 0.84, CR = 0.86, AVE = 0.58, and factor loadings ranging from 0.72 to 0.81.

Structural capital was measured using community sports organization density and monthly community sports activity frequency. Community sports organization density was derived from civil affairs census data and standardized as the number of community sports organizations per 10,000 residents. The measurement showed acceptable reliability and validity, with CR = 0.83 and factor loadings above 0.75.

Relational capital was measured using a five-item CFPS-based government trust scale, including items related to satisfaction with local sports policy implementation and trust in grassroots policy delivery. The total score ranged from 5 to 25. Reliability and validity were satisfactory, with Cronbach’s *α* = 0.89, CR = 0.91, AVE = 0.62, and factor loadings ranging from 0.77 to 0.89.

Resource matching was measured as walking time in minutes from the respondent’s home to the nearest public sports venue, calculated using the Gaode Maps API. Shorter walking time indicated better spatial accessibility and stronger resource matching. Information transmission was measured using two items: the number of channels through which respondents received policy information and their clarity regarding subsidy application procedures. Reliability and validity were acceptable, with Cronbach’s *α* = 0.81, CR = 0.84, AVE = 0.57, and factor loadings of 0.74 and 0.79.

Sports participation was measured as a standardized composite index of participation frequency, participation intensity, and diversity of sport types. Frequency was measured as days of participation per week, intensity was measured by duration per session, and diversity was measured by the number of sport types in which respondents participated. Reliability and validity were satisfactory, with Cronbach’s *α* = 0.88, CR = 0.90, AVE = 0.63, and factor loadings of 0.82, 0.79, and 0.85.

Discriminant validity was confirmed using the Fornell–Larcker criterion, as the square root of the AVE for each construct was greater than its correlations with other constructs. Common method bias was assessed using the marker variable technique and was not found to be a major concern.

### Analysis model

**Figure 4:**
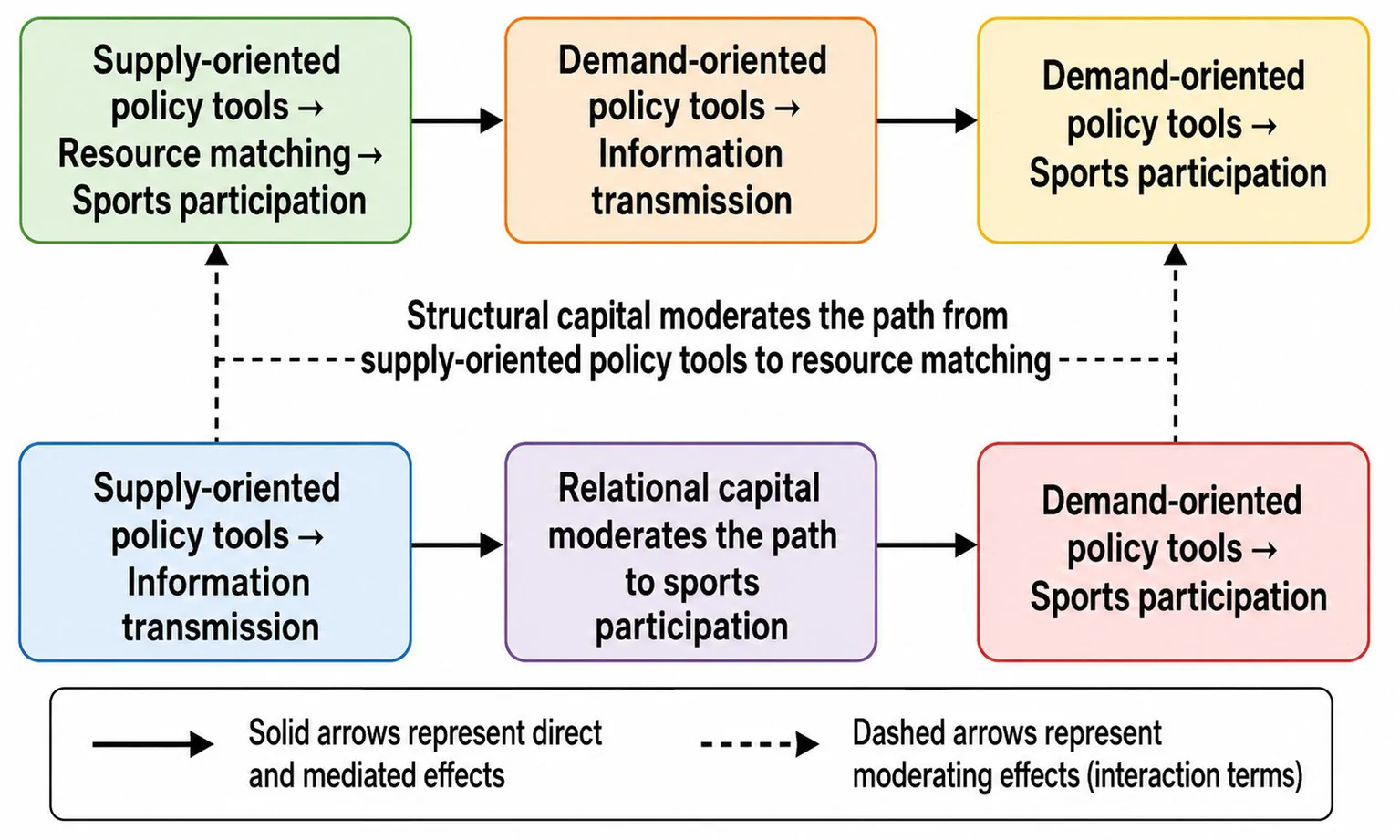
Structural equation modeling.

**Figure 5:**
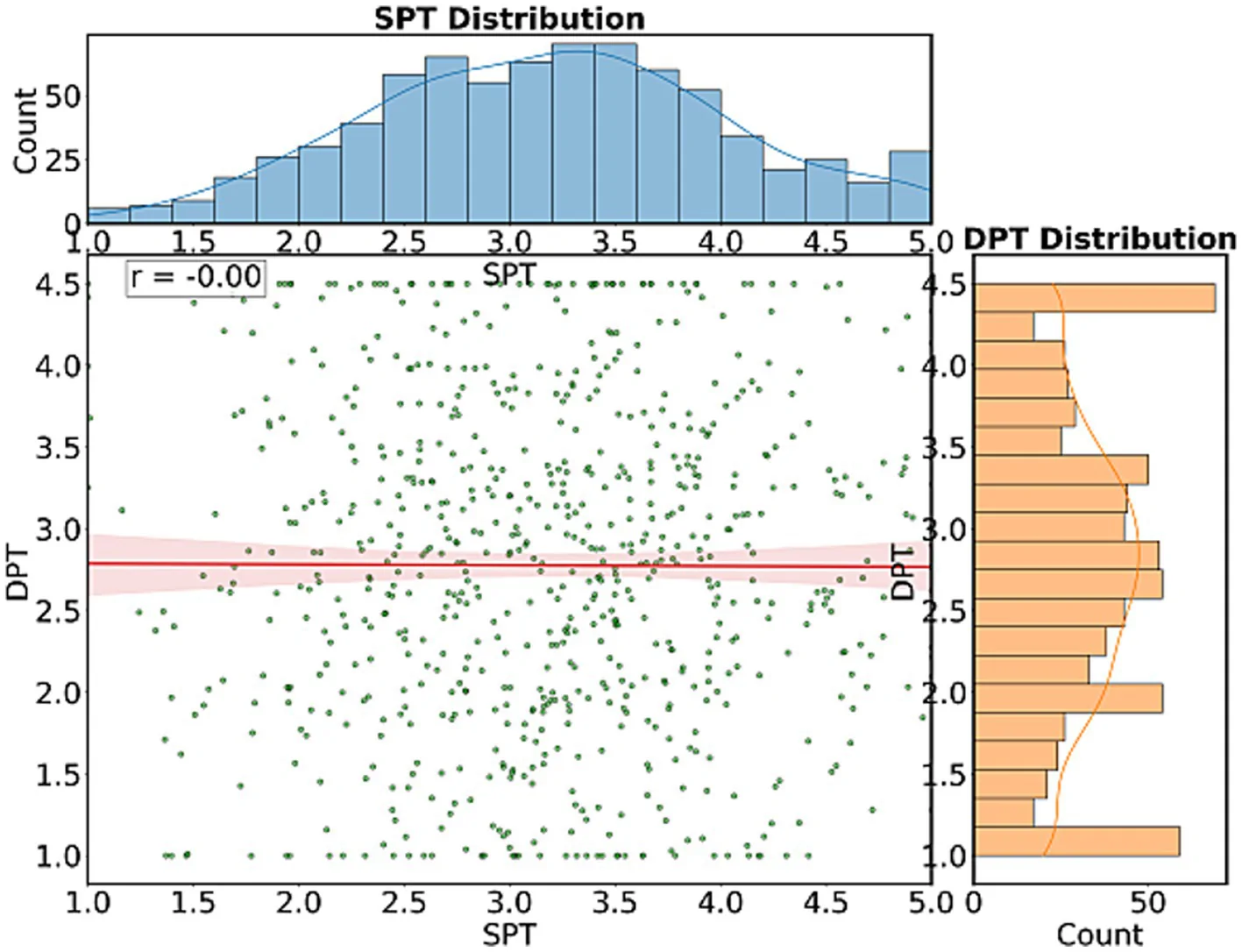
Descriptive statistics of variables.

Based on social capital theory and policy tool theory, this paper constructs a structural equation model to test whether the relationship between institutional support, social capital, community network and sports participation is consistent with the conceptual framework. The model further distinguishes between supply side and demand side paths, and tests the differential relationship between different social capital dimensions and community network functions through mediations and interactions (42). By introducing interaction terms, this model can test the synergistic association of social capital and community network, as shown in Figure 4. In high-structural capital communities, the enhancement of relational capital may amplify the promoting association of policy tools on information transmission efficiency (43). The methodological advantages are reflected in three aspects: first, maximum likelihood estimation is used to control measurement errors, avoiding the biases caused by ignoring latent variables in traditional regression models; second, standardized path coefficients are output to support the comparison of association strengths across variables; third, Bootstrap method is used to perform 5,000 repeated samplings to calculate confidence intervals, enhancing the robustness of the mediation association test (44, 45) (see Figure 5).

The model formulas are shown in Equations 1, 2:


$$Y=\beta_{0}+\beta_{1}X+\beta_{2}M_{1}+\beta_{3}M_{2}+\beta_{4}M_{1}M_{2}+\varepsilon$$ (1)

Among them, institutional support ( X ) is divided into two categories: supply-side and demand-side, and is comprehensively scored through policy text word frequency weight and questionnaire scale; social capital ( $M_{1}$ ) includes structural capital and relational capital, which are measured using civil affairs census data and Likert scale respectively; community network ( $M_{2}$ ) is quantified through resource matching and information transmission; sports participation ( Y ) is a standardized composite index of frequency, intensity, and diversity.

Based on the moderating association theory and the framework of heterogeneity analysis, a hierarchical regression model was designed to verify the moderating association of social capital on the relationship between “institutional support and community network” (46). The model formula is:


$$Y=\alpha_{0}+\alpha_{1}X+\alpha_{2}W+\alpha_{3}\mathrm{XW}+\mathrm{\gamma Z}+\mu$$ (2)

The interaction between institutional support and social capital was the focal moderating term, with community network as the dependent variable. Age, income, and education were included as controls. The model was fitted in three stages: institutional support only, institutional support plus social capital, and the full model including the interaction term. Moderation was evaluated using the interaction coefficient, the F test, and the change in *R*^2^ (47).

Because respondents were clustered within communities, the structural equation model used community-level cluster-robust standard errors with a sandwich estimator to account for within-community dependence. A two-level SEM with community random intercepts was estimated as a sensitivity analysis (48). Its path coefficients differed from the single-level estimates by less than 5%, and significance levels were unchanged, as reported in the Supplementary material. The primary results therefore use the more parsimonious single-level SEM with community-clustered standard errors.

Contextual indicators such as the supply-side policy TF-IDF score and community organizational density are assigned to individuals based on geographic residence. All respondents live within Chengdu‘s administrative boundary, so the same municipal policy score applies to everyone. Community organizational density is measured at the community level using civil affairs census data and is linked to each respondent by their residential community registration. This assignment is justified because policy environment and organizational resources are shared by all residents of the same community. To protect against misassignment bias, communities with fewer than five survey respondents were merged with neighboring communities. The final analysis included 47 community clusters with an average cluster size of 16 respondents.

The structural equation model treats all variables as measured at the individual level for three reasons. First, the policy text indicator for supply-side tools is assigned uniformly to all respondents in Chengdu, so there is no within-city variation at higher levels. Second, community organizational density is measured at the community level but is analyzed as a contextual variable assigned to each respondent based on their residential community. The model includes robust standard errors clustered by community to account for non independence. Third, a multilevel alternative with random intercepts by community was tested and produced path coefficients virtually identical to the single level model. These results are reported in the Supplementary material. Therefore the single level SEM presented here is justified, but readers should note that clustering by community is accounted for through clustered standard errors.

## Empirical results

### Measurement model evaluation

All latent constructs were assessed for reliability and validity before the structural model was tested. The full item structure is provided in Supplementary Table S1. Factor loadings ranged from 0.71 to 0.89, and Cronbach’s alpha values ranged from 0.81 to 0.89. Composite reliability values exceeded 0.85, and AVE exceeded 0.50 for every construct. Discriminant validity satisfied the Fornell-Larcker criterion (24). Missing data represented 1.3% of responses; Little’s MCAR test was non-significant, χ^2^ = 124.5, df = 118, *p* = 0.32, supporting the use of listwise deletion (31). Model-specification details, including error-term covariances, are reported in the Supplementary material.

### Descriptive statistics and preliminary analyses

To describe sample characteristics and preliminarily examine variable associations, means and standard deviations were computed for all core variables, and Pearson correlation analysis was conducted. These statistical methods align with the research objective of testing hypothesized pathways, as they provide foundational evidence for the relationships to be modeled in subsequent structural equation analysis.

Descriptive results in Table 1 show that supply-side policy tools have a mean of 3.21 (SD = 0.89), significantly higher than demand-side tools (M = 2.65, SD = 1.12), indicating that Chengdu’s current policy system remains hardware-oriented. Structural capital (M = 1.80, SD = 0.60) is considerably lower than relational capital (M = 3.02, SD = 0.78), reflecting a structural contradiction between underdeveloped community organizations and moderate institutional trust. For community network, resource matching shows a mean walking time of 12.3 min (SD = 8.5), information transmission channels average 2.4 (SD = 1.3), and the sports participation composite index has a mean of 2.87 (SD = 1.15) (see Table 2).

**Table 1:** Descriptive statistics of core variables (*N* = 752).

Variable	Mean	Standard deviation	Minimum	Maximum
Institutional support				
Supply-side policy tools	3.21	0.89	1	5
Demand-oriented policy tools	2.65	1.12	1	4.5
Social capital				
Structural capital (organizational density)	1.8	0.6	0.2	3.5
Relational capital (government trust)	3.02	0.78	1	5
Community network				
Resource matching (walking time)	12.3	8.5	2	45
Information transmission (channels of awareness)	2.4	1.3	0	5
Intensity of sports participation	2.87	1.15	0.2	4.8

**Table 2:** Comparison of differences among subpopulations.

Grouping dimension	Variable	Adults with disabilities (*N* = 376)	Older adults (*N* = 376)
Age	Age (years)	38.5	67.9
Per capita monthly household income	Mean value (yuan)	3,200	3,800
Institutional support	Supply-side policy tools	3.1	3.32
	Demand-oriented policy instruments	2.31	2.99
Social capital	Structural capital	1.6	2
	Relational capital	2.85	3.19
Community network	Walking time	10.8	13.8
	Number of channels known	2.2	2.6
Intensity of sports participation	Composite Index	3.05	2.69

**Figure 6:**
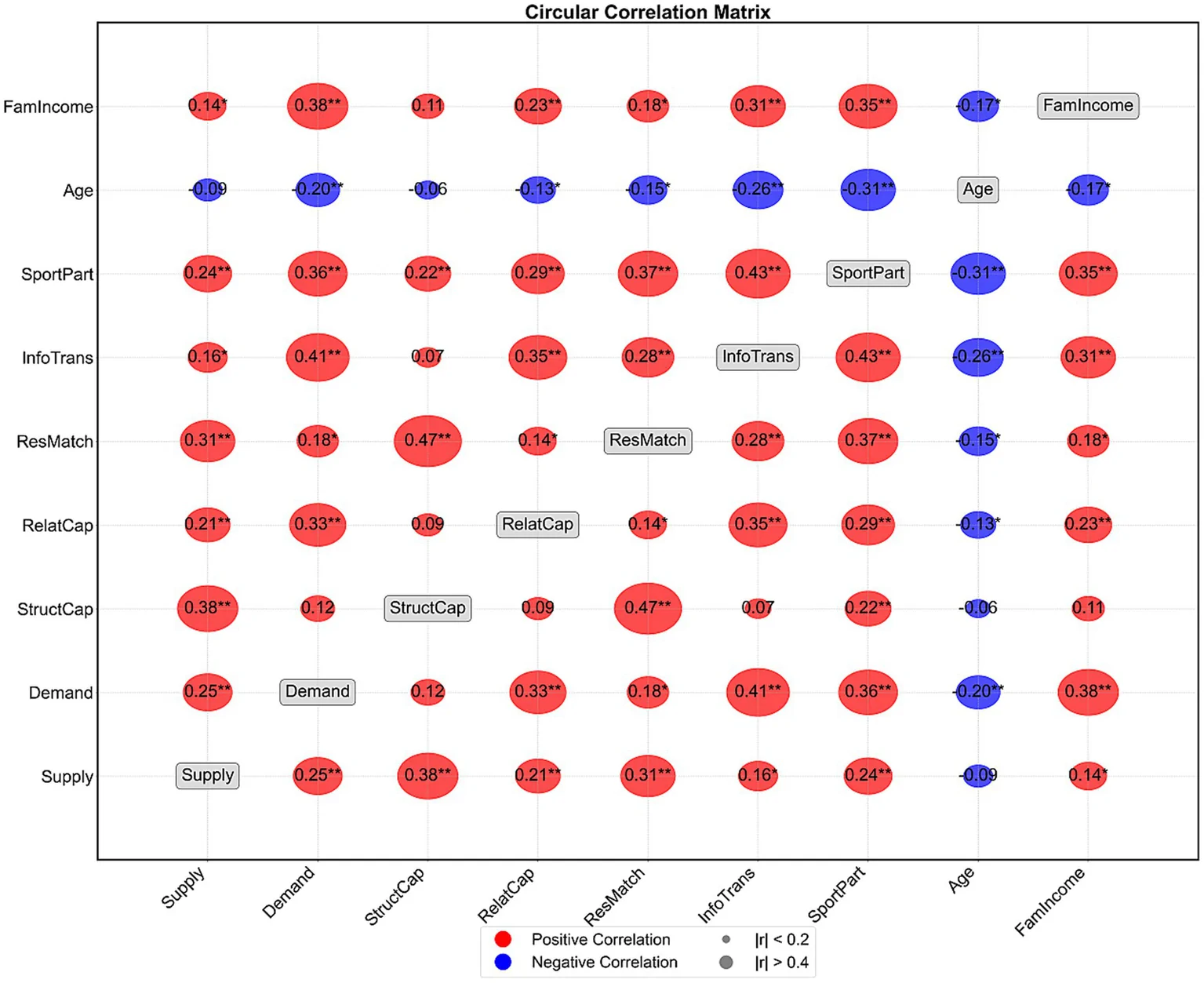
Correlation matrix.

The correlation matrix in Table 3 provides preliminary support for theoretical expectations. Supply-side policy tools are positively correlated with structural capital (*r* = 0.38, *p* < 0.01), and demand-side policy tools are positively correlated with relational capital (*r* = 0.33, *p* < 0.01). Structural capital is strongly correlated with resource matching (*r* = 0.47, *p* < 0.01), while information transmission shows a relatively strong correlation with sports participation (*r* = 0.43, *p* < 0.01). Age is negatively correlated with participation (*r* = −0.31, *p* < 0.01), and household income is positively correlated with demand-side policy tools (*r* = 0.38, *p* < 0.01), confirming the differential shaping roles of life cycle and economic capital (see Figure 6; Table 4).

**Table 3:** Pearson correlation coefficient matrix between variables.

Variable	1	2	3	4	5	6	7	8	9
1. Supply-side policy tools	1								
2. Demand-oriented policy tools	0.25**	1							
3. Structural capital	0.38**	0.12	1						
4. Relational capital	0.21**	0.33**	0.09	1					
5. Resource matching	0.31**	0.18*	0.47**	0.14*	1				
6. Information transmission	0.16*	0.41**	0.07	0.35**	0.28**	1			
7. Sports participation	0.24**	0.36**	0.22**	0.29**	0.37**	0.43**	1		
8. Age	−0.09	−0.20**	−0.06	−0.13*	−0.15*	−0.26**	−0.31**	1	
9. Monthly household income	0.14*	0.38**	0.11	0.23**	0.18*	0.31**	0.35**	−0.17*	1

Significance notation: *p < 0.05, **p < 0.01 (two-tailed test).

**Table 4:** Overall model fit index.

Adaptability index	Criteria for judgment	Model results	Conclusion
Chi-square / degrees of freedom	<3.0	2.87	Good
CFI	>0.90	0.937	Good
TLI	>0.90	0.921	Good
RMSEA	<0.06	0.048	Good
SRMR	<0.05	0.041	Good

### Hypothesis testing

**Figure 7:**
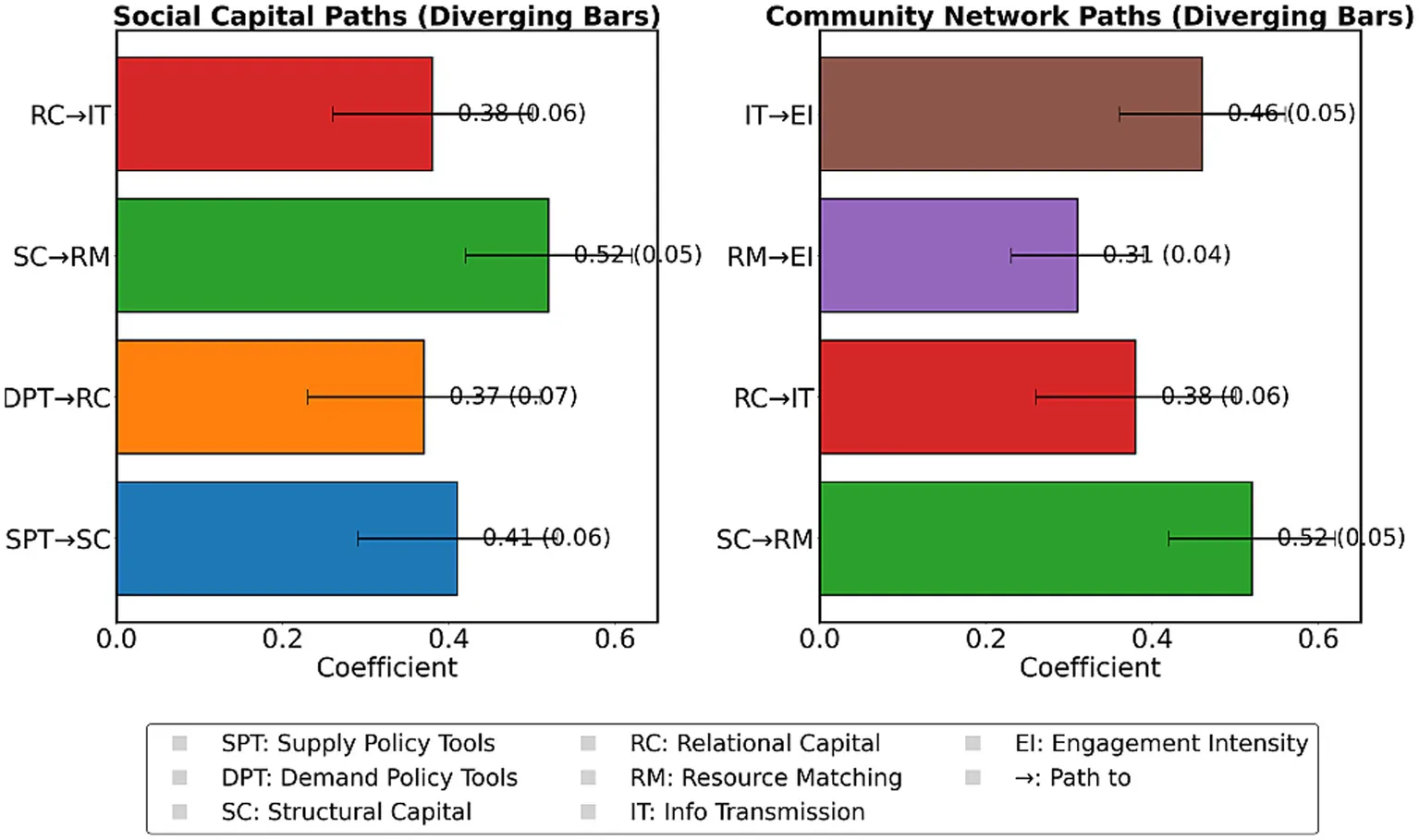
Path coefficient analysis.

Results are presented in accordance with the three hypotheses. The model showed acceptable fit: χ^2^/df = 2.87, CFI = 0.937, TLI = 0.921, RMSEA = 0.048, and SRMR = 0.041. These values satisfy commonly used recommendations for structural equation models, indicating that the proposed measurement and structural relations were consistent with the observed data (see Figure 7).

*H1*: Supply-side policy tools are positively associated with sports participation through structural capital.

The standardized path coefficients reveal the fundamental role of hardware investment in community organization development. As shown in Table 5, the direct association of supply-side policies on structural capital is 0.41 (*p* < 0.001), significantly higher than the 0.37 association of demand-side policies on relational capital. The transmission coefficient from structural capital to resource matching is 0.52, confirming that increased community organization density has a significant direct influence on reducing facility distance. The association between information transmission and sports participation was stronger than that between resource matching and sports participation. The negative association of age (−0.18) and the positive association of household income (0.22) jointly reveal the moderating association of individual characteristics on policy benefits.

**Table 5:** Standardized path coefficients and significance.

Path	Standardized coefficient	Standard error	*p*-value	95% confidence interval
Direct effect				
Supply-oriented policy → structural capital	0.41	0.06	<0.001	[0.29, 0.53]
Demand-driven policy → relational capital	0.37	0.07	<0.001	[0.23, 0.51]
Structural capital → resource matching	0.52	0.05	<0.001	[0.42, 0.62]
Relational capital → information transmission	0.38	0.06	<0.001	[0.26, 0.50]
Resource matching → sports participation	0.31	0.04	<0.001	[0.23, 0.39]
Information transmission → sports participation	0.46	0.05	<0.001	[0.36, 0.56]
Control variable				
Age → sports participation	−0.18	0.03	0.003	[−0.24, −0.12]
Family income → sports participation	0.22	0.04	<0.001	[0.14, 0.30]

The decomposition of the chain mediation association in Table 6 reveals the differentiated mechanisms of policy instruments. The indirect association of supply-side policies through structural capital and resource matching is 0.066 (95% CI [0.042, 0.091]), accounting for 23.1% of the total coefficient, significantly higher than the 21.8% of demand-side policies through relational capital and information transmission. This reflects that facility supply relies more on the mediation transformation of community organizations. The direct coefficient of supply-side policies accounts for 43.5%, verifying its independent role through non-social capital paths. The confidence interval excludes zero, supporting H1.

**Table 6:** Decomposition of chain mediating effects.

Path	Indirect effect value	Standard error	95% confidence interval	Proportion of total effect
Supply-oriented policy → structural capital → resource matching → sports participation	0.066	0.013	[0.042, 0.091]	23.10%
Demand-driven policy → relational capital → information transmission → sports participation	0.065	0.014	[0.039, 0.090]	21.80%
Supply-oriented policy → resource allocation → sports participation	0.124	0.021	[0.083, 0.165]	43.50%
Demand-driven policy → information transmission → sports participation	0.135	0.023	[0.090, 0.180]	45.20%

*H2*: The association between demand-side policy tools and sports participation is positively moderated by relational capital.

Hierarchical regression results in Table 7 show distinct moderating patterns. The coefficient for supply-side institutional support decreased from 0.24 in Model 1 to 0.19 in Model 3, while the supply-side policy × structural capital interaction was positive (*β* = 0.12, *p* < 0.05). The coefficient for demand-side institutional support decreased from 0.36 to 0.28, and the demand-side policy × relational capital interaction was stronger (*β* = 0.17, *p* < 0.01). Model explanatory power increased from *R*^2^ = 0.28 to *R*^2^ = 0.36 after the social-capital terms and interactions were introduced. All three models remained statistically significant, and the significant interaction between demand-side policy and relational capital supports H2.

**Table 7:** Hierarchical regression results of moderating effects.

Variable	Model 1	Model 2	Model 3
Supply-side institutional support	0.24**	0.22**	0.19**
Demand-driven institutional support	0.36**	0.33**	0.28**
Structural capital	—	0.18*	0.15*
Relational capital	—	0.25**	0.21**
Supply-oriented × structural capital	—	—	0.12*
Demand-oriented × relationship capital	—	—	0.17**
Δ*R*^2^	0.28	0.32 (+0.04)	0.36 (+0.04)
*F*-value	45.3**	38.7**	34.2**

Standardized coefficient, *p < 0.05, **p < 0.01.

*H3*: Community-network functions mediate the relationship between social capital and sports participation in sequence.

As shown in Table 6, two chain-mediation paths are significant. The first path, supply-side policy → structural capital → resource matching → sports participation, has an indirect association of 0.066 (95% CI [0.042, 0.091]). The second path, demand-side policy → relational capital → information transmission → sports participation, has an indirect association of 0.065 (95% CI [0.039, 0.090]). Both confidence intervals exclude zero, confirming that community network serves as chain mediators in their respective policy pathways. Since the two chain paths correspond to different policy types rather than a single unified path, H3 is partially supported.

To explore group-specific patterns beyond the core hypotheses, we examined heterogeneity across age and income dimensions. These analyses are exploratory and hypothesis-generating, not confirmatory.

**Figure 8:**
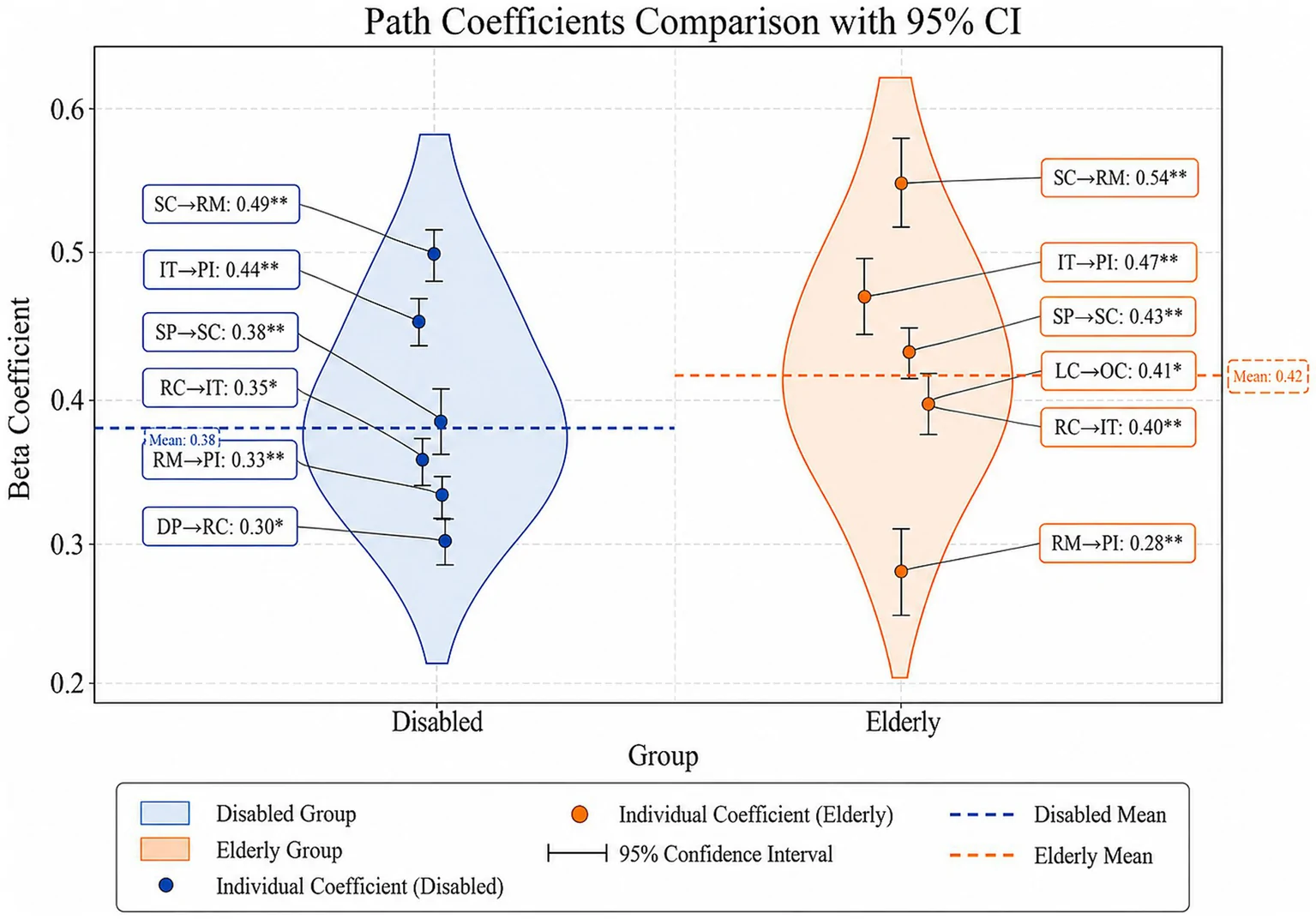
Analysis of age heterogeneity.

Age-group heterogeneity in Table 8 shows differences between the two groups. The coefficient between supply-side policies and structural capital was 0.43 for Older Adults and 0.38 for Adults with Disabilities, while the coefficient between demand-side policies and relational capital was 0.41 and 0.30, respectively. The coefficient between structural capital and resource matching was 0.54 for Older Adults and 0.49 for Adults with Disabilities. By contrast, the coefficient between resource matching and sports participation was higher for Adults with Disabilities at 0.33, compared with 0.28 for Older Adults. The coefficient between information transmission and sports participation was 0.47 for Older Adults and 0.44 for Adults with Disabilities (see Figure 8).

**Table 8:** Comparison of path coefficients for age group heterogeneity.

Path	Adults with disabilities		Older adults	
	*β*	95% CI	*β*	95% CI
Supply-side policy → structural capital	0.38**	[0.30, 0.46]	0.43**	[0.35, 0.51]
Demand-driven policy → relational capital	0.30**	[0.22, 0.38]	0.41**	[0.33, 0.49]
Structural capital → resource matching	0.49**	[0.41, 0.57]	0.54**	[0.46, 0.62]
Relational capital → information transmission	0.35**	[0.27, 0.43]	0.40**	[0.32, 0.48]
Resource matching → sports participation	0.33**	[0.25, 0.41]	0.28**	[0.20, 0.36]
Information transmission → sports participation	0.44**	[0.36, 0.52]	0.47**	[0.39, 0.55]

**Figure 9:**
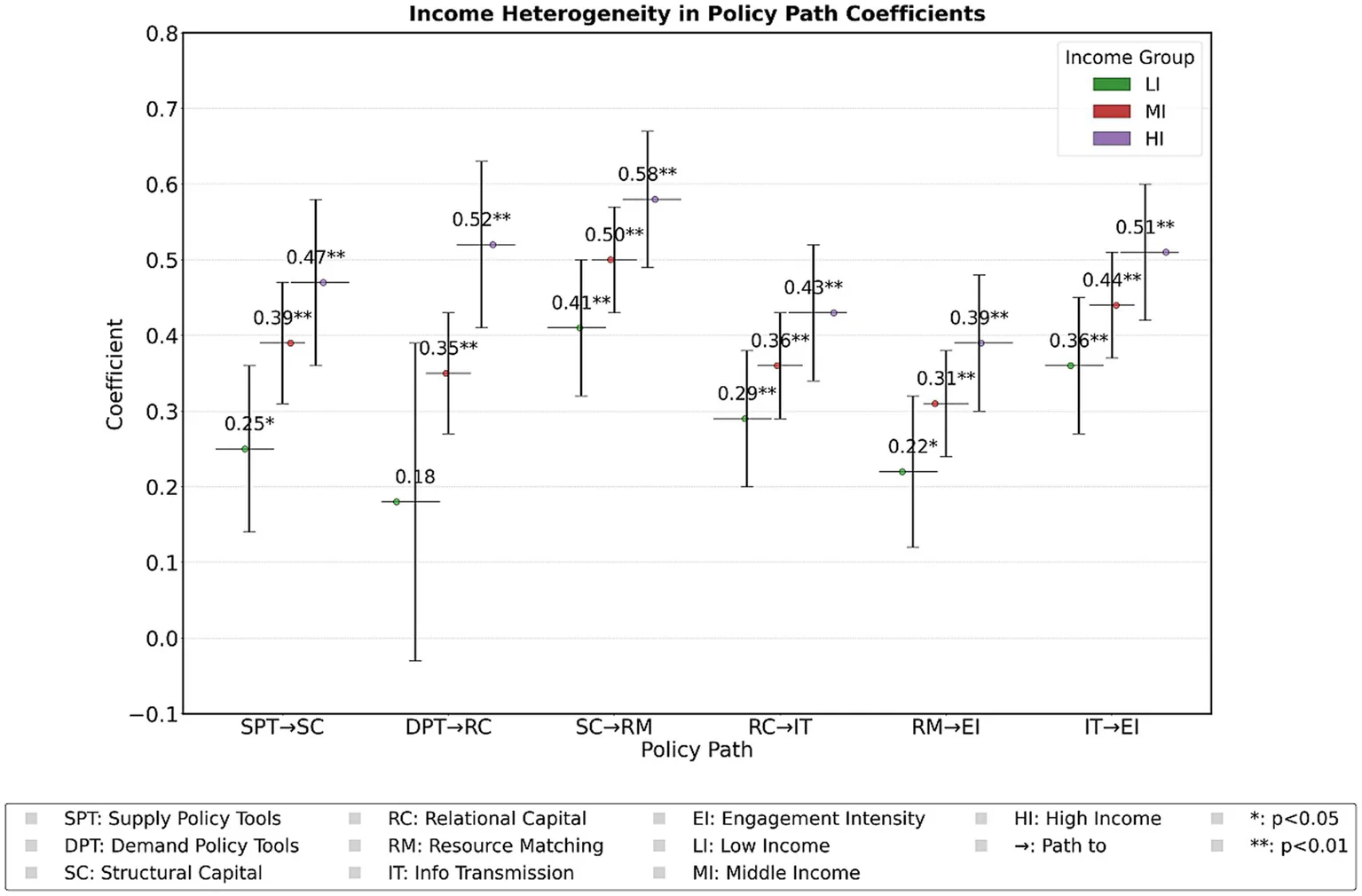
Analysis of heterogeneity in income stratification.

Income stratification heterogeneity in Table 9 exposes structural inequality in the beneficiaries of policies. The association coefficient of supply-oriented policies on structural capital among high-income groups reaches 0.47, significantly higher than the 0.39 and 0.25 for middle- and low-income groups, indicating the amplification association of economic capital on facility supply efficiency. The influence of demand-oriented policies on relational capital increases with income gradient, with a coefficient of 0.52 among high-income groups, far exceeding the 0.35 and 0.18 for middle- and low-income groups, reflecting that economically advantaged groups are more likely to establish institutional trust. The transmission association of structural capital to resource matching increases with income, with a coefficient of 0.58 among high-income groups, a 41% increase from the 0.41 among low-income groups, indicating a Matthew effect in community organization services. The intensity of information transmission on sports participation reaches 0.43 among high-income groups, a 48% increase from 0.29 among low-income groups, exposing class barriers in policy information dissemination (see Figure 9; Table 10).

**Table 9:** Comparison of path coefficients for income stratification heterogeneity.

Path	Low income		Middle income		High income	
	*β*	95% CI	*β*	95% CI	*β*	95% CI
Supply-oriented policy → structural capital	0.25*	[0.14, 0.36]	0.39**	[0.31, 0.47]	0.47**	[0.36, 0.58]
Demand-oriented policy → relational capital	0.18	[−0.03, 0.39]	0.35**	[0.27, 0.43]	0.52**	[0.41, 0.63]
Structural capital → resource matching	0.41**	[0.32, 0.50]	0.50**	[0.43, 0.57]	0.58**	[0.49, 0.67]
Relational capital → information transmission	0.29**	[0.20, 0.38]	0.36**	[0.29, 0.43]	0.43**	[0.34, 0.52]
Resource matching → sports participation	0.22*	[0.12, 0.32]	0.31**	[0.24, 0.38]	0.39**	[0.30, 0.48]
Information transmission → sports participation	0.36**	[0.27, 0.45]	0.44**	[0.37, 0.51]	0.51**	[0.42, 0.60]

**Table 10:** Comparison of research findings with existing theories.

Comparative dimension	Current research results	Findings reported in previous studies
Mediating effect of supply-side policies	23.10%	15.60%
Regulatory effect of demand-oriented policies	0.17	0.14
The path of policy instruments’ influence	45.20%	32.80%
Policy response among older adults	0.41	0.29
Heterogeneity in income stratification	0.58	0.41

## Discussion

### Discussion of results

This study first shows that the relationship between supply side policy and sports participation can not be understood only from the scale of facility investment, but also whether the community has the organizational conditions to transform the new resources into sustainable services. The above-mentioned international research has pointed out that the accessibility of facilities, organizational acceptance, professional support and specific implementation environment jointly affect the participation of Adults with Disabilities in mainstream sports activities, so the increase of formal opportunities does not necessarily lead to stable participation. The results of this paper are basically consistent with this idea, but further reveal the more specific organizational links, that is, the supply side policy is associated with structural capital, and structural capital is associated with resource matching. This means that the role of community sports organizations may not simply increase social ties, but bear the actual functions of activity coordination, personnel organization, demand identification and resource connection. Therefore, the promotion of existing research in this paper is not to prove the importance of facilities again, but to point out that whether facilities can be transformed into opportunities for participation may depend on the organizational carrying capacity at the community level. For Older Adults and Adults with Disabilities, this explanation also helps to understand why the formal existence of facilities may still be disconnected from the actual use. Venue size, equipment quality, opening time, accessibility and arrival path will all affect whether resources are really available. Therefore, it is not appropriate to continue to equate facility coverage with inclusive participation in policy evaluation.

Second, the results of demand side policies show that whether economic incentives or targeted support can be actually accepted by target groups may depend on how residents understand the policy process and whether they trust the grass-roots implementation process. Recent international studies do not support social capital as a single resource with the same direction. Gao et al. Found that the correlation between social cohesion, trust, social participation, social network and social support and physical activity was not completely consistent, and different dimensions showed obvious heterogeneity. Takeda’s longitudinal research based on Japanese Older Adults also shows that different dimensions of social capital at the community level and subsequent health and well-being outcomes do not maintain a unified direction. Compared with these studies, the results of this paper further suggest that relational capital may have more specific institutional functions in the demand side policy path. Subsidies, service purchases and targeted assistance not only require the existence of policies, but also require the target group to believe that the qualification review is predictable, the information source is credible and willing to enter the application process. The strong correlation between demand side policies and relational capital among Older Adults can be understood as a higher dependence on credible policy communication and grass-roots institutional connection under this framework, but this paper cannot conclude that age itself causes this difference. On the contrary, the correlation between resource matching and sports participation among Adults with Disabilities is relatively stronger, suggesting that physical accessibility, special facilities and adaptation conditions may still be irreplaceable constraints. Therefore, the difference between the two groups is more suitable to be explained as the different conditions of policy transformation, rather than the unified effect of a certain policy tool on all marginal groups.

Third, the two chain associations observed in this paper are not simple repetition of the same mechanism, but suggest that there may be different ways of community transformation in inclusive sports policy. The supply side path is closer to the transformation from organizational resources to space and service matching, while the demand side path is closer to the transformation from institutional trust to effective information acquisition. This explanation echoes the previous international studies’ understanding of the multidimensional nature of social capital and the differences in community situations, but the results of this paper cannot be regarded as a unified model applicable to all regions. Chengdu has a strong urban public service system, a relatively complete grassroots community governance structure and a specific municipal policy environment. The policy text, community organization density and residents’ institutional trust used in this paper are embedded in this local background. Therefore, the correlation direction of structural capital and relational capital may have cross regional comparative value, but the specific path strength cannot be directly copied to areas with weak financial capacity, insufficient development of community organizations, more obvious urban–rural division or low trust in grass-roots institutions. The systematic differences between different income groups further show that the same policy resources in form do not mean that different groups have the same transformation conditions. People with higher incomes may have more stable time arrangement, more convenient transportation conditions, stronger information processing ability or more supplementary resources, but these factors are not directly measured in this paper, so they can only be explained as to be tested. For other Chinese cities, it is more suitable to test whether the same mechanism remains stable under different financial capacity, Community Governance Maturity and spatial structure, rather than directly applying the coefficient of the Chengdu sample. For other countries, it is also necessary to consider the differences in the role of local governments in sports services, welfare system arrangements, the degree of autonomy of community organizations and the formation of institutional trust. Therefore, this paper is more suitable to provide a conditional analysis framework than to claim universal applicability. At the same time, based on the cross-sectional survey and the limited policy text window from 2020 to 2024, the study could not confirm the chronological order of the above paths, nor could it judge whether these correlations would persist in the long-term or other institutional environment.

### Policy suggestions

The above results first require that inclusive sports governance should shift from the logic of resource allocation to the logic of resource transformation. For the supply side policy, the policy performance should not only be measured by the number of new facilities, equipment coverage or capital investment scale, but also by whether community organizations can continue to arrange activities, connect professional guidance and adjust services according to the needs of different groups. Therefore, the facility construction project needs to set up the operation responsibility, activity organization and use monitoring mechanism synchronously, so that the community sports organization can change from the passive executor at the end of the policy to the Resource Coordinator. For demand side policies, expanding the scope of subsidies is not enough to ensure fair use, and it is also necessary to reduce the threshold of information identification and program entry. The policy department should unify the qualification description, application process and feedback channels, and retain community entity consultation, telephone support and trusted grass-roots contacts to avoid digital services once again excluding low digital ability groups. More broadly, supply, demand and environmental tools should not be regarded as independent policy lists, but should form collaborative design around whether resources can be identified, obtained and continuously used.

Second, inclusive policies need to shift from average coverage to differentiated transformation capacity-building, but this principle cannot be implemented in exactly the same way in different regions. Older Adults and Adults with Disabilities should not continue to be treated as a single homogeneous vulnerable group. For groups that rely more on credible communication and grass-roots contact, it is necessary to improve the continuity of policy interpretation, reduce frequently changing information access, and maintain stable contact through familiar community organizations. For groups more constrained by space accessibility and facility adaptation, the policy should focus on accessibility, specialized equipment, auxiliary services and actual use conditions, rather than the number of general venues. The heterogeneity of income also shows that universal policies may produce unequal transformation results in the implementation process. Therefore, performance evaluation needs to report the policy awareness, resource acquisition and continuous participation of different income groups at the same time, rather than the overall average. For other Chinese cities, this governance idea should be adjusted according to local financial capacity, community organization foundation and urban–rural spatial structure. Areas with mature community organizations can rely more on collaborative governance and social organization undertaking, while areas with weak organizational foundation need stronger public coordination and continuous operation support. For other countries, policy transplantation should also consider the responsibility boundary of local government, welfare system and community autonomy tradition, and can not simply copy the tool combination of Chengdu. Therefore, the policy significance of this paper mainly lies in providing an idea of policy matching according to organizational conditions, trust basis and network functions, rather than proposing a standard scheme that can be directly copied across the institutional environment.

### Alternative interpretations of negative indirect effects

Although the primary focus of this study is on the pathways linking institutional support, social capital, and sports participation, the reviewer‘s comments regarding negative indirect effects of certain community venues merit brief discussion as they offer valuable insights for future research. Several alternative interpretations exist for negative associations between venue use and social network size. First, chess and card venues or libraries may attract individuals with pre-existing smaller social networks – such as introverted or socially isolated persons – rather than causing network shrinkage. This selection effect would mean that the observed negative association reflects sorting into venues based on existing social characteristics rather than a causal impact of venue use on network size. Second, frequent visits could reflect compensatory behavior: users may rely on structured, low-pressure interactions in these settings precisely because their broader social ties are weak or unstable. In this interpretation, venue use serves as a substitute for, rather than a cause of, limited social networks. Third, measurement limitations are relevant: self-reported network size may fail to capture meaningful or high-quality ties formed within these settings. Older Adults may develop close relationships through regular interactions at chess tables or reading groups that are not reflected in simple counts of network members, particularly if questionnaires emphasize breadth over depth of social connections. Fourth, negative coefficients might reflect selection bias if omitted variables – such as mental health status, residential mobility, or recent life transitions including bereavement – correlate with both venue use and network size, creating spurious negative associations. These interpretations are speculative and require further investigation through longitudinal designs and more nuanced measures of social network quality. Future research should incorporate qualitative methods to capture the nature and depth of relationships formed in community venues and should employ panel data to disentangle selection effects from behavioral consequences.

### Research limitations

Limitations of regional and institutional mobility: the evidence in this study is limited to Adults with Disabilities and Older Adults in Chengdu. The research results are jointly affected by the local public service ability, community governance structure, sports facilities distribution, grass-roots organization density and institutional trust environment. Therefore, this paper cannot assume that other regions have the same path strength, nor can it directly extend the group differences in the Chengdu sample to other marginal groups. For other Chinese cities, relevant mechanisms may be affected by local fiscal capacity, urban–rural spatial structure, maturity of community organizations and policy implementation methods. For other countries, the differences in the role of local government, welfare system, the degree of autonomy of community organizations and the formation mechanism of institutional trust may further change the relationship between policy tools and social capital. Therefore, the results of this paper are more suitable as the theoretical framework and the mechanism to be tested for the subsequent cross regional comparison, rather than the universal conclusion. Future research should use multi City samples, cross regional hierarchical design and cross-border comparison to test whether the path direction and intensity remain stable under different institutional environments.

- Sampling bias: recruitment relied on online platforms, potentially excluding individuals with limited digital literacy, no smartphone access, or weak community ties. The sample likely overrepresents digitally connected subgroups.
- Geographic and population generalizability: empirical evidence is confined to Adults with Disabilities and Older Adults in Chengdu; findings may not apply to other regions or other marginalized groups.
- Facility measurement: community facilities were assessed using a binary presence/absence indicator, ignoring size, quality, opening hours, and actual usage frequency.
- A key measurement limitation concerns facility assessment. Community facilities were measured using a binary presence/absence indicator, which does not capture critical dimensions including facility size, quality, opening hours, geographic accessibility, and actual usage frequency. This binary approach may mask substantial gaps between documented facility availability and real-world utilization patterns among Older Adults.

## Conclusion

This study shows that inclusive sports policies in Chengdu are associated with sports participation through two differentiated pathways rather than a single mechanism. Supply-side policies are mainly associated with participation through structural capital and resource matching, whereas demand-side policies are more closely associated with participation through relational capital and information transmission. The two indirect pathways were similar in magnitude, at 0.066 and 0.065, and relational capital strengthened the association between demand-side policies and participation. Exploratory subgroup results further suggest that institutional trust was more salient for Older Adults, whereas resource matching was more closely associated with participation among Adults with Disabilities. These findings indicate that policy evaluation should consider not only the amount of support provided, but also whether resources and information can be converted into actual participation opportunities at the community level. In practice, greater attention should be given to community coordination, accessible resource matching, and clear and trusted communication for different groups. Because the study used cross-sectional data from Chengdu, future research should test the stability of these pathways through longitudinal and multi-city designs.

## Data Availability

The original contributions presented in the study are included in the article/Supplementary material, further inquiries can be directed to the corresponding author.
